# Bilateral Pheochromocytoma with Germline MAX Variant without Family History

**DOI:** 10.3390/clinpract12030035

**Published:** 2022-05-07

**Authors:** Shinnosuke Hata, Mai Asano, Hiroyuki Tominaga, Masahide Hamaguchi, Fumiya Hongo, Takeshi Usui, Eiichi Konishi, Michiaki Fukui

**Affiliations:** 1 Department of Endocrinology and Metabolism, Graduate School of Medical Science, Kyoto Prefectural University of Medicine, Kyoto 602-8566, Japan; hatashin@koto.kpu-m.ac.jp (S.H.); htommy@koto.kpu-m.ac.jp (H.T.); mhama@koto.kpu-m.ac.jp (M.H.); michiaki@koto.kpu-m.ac.jp (M.F.); 2 Department of Endocrinology and Metabolism, Kyoto First Red Cross Hospital, Kyoto 605-0981, Japan; 3 Department of Urology, Graduate School of Medical Science, Kyoto Prefectural University of Medicine, Kyoto 602-8566, Japan; fhongo@koto.kpu-m.ac.jp; 4 Research Support Center, Shizuoka General Hospital, Shizuoka 420-8527, Japan; tusui1220@gmail.com; 5 Department of Medical Genetics, Shizuoka General Hospital, Shizuoka 420-8527, Japan; 6 Department of Pathology, Kyoto Prefectural University of Medicine, Kyoto 602-8566, Japan; konie@koto.kpu-m.ac.jp

**Keywords:** MYC-associated factor X gene, multiple pheochromocytoma, bilateral pheochromocytoma, sporadic pheochromocytoma, adrenal tumor

## Abstract

Recently, the genetic background of pheochromocytomas/paragangliomas (PPGLs) has been rapidly revealed. These tumors have been referred to as the “ten percent tumor”; however, the frequency of genetic variants of PPGLs has turned out to be more common than expected. PPGLs are potentially hereditary tumors and appear clinically sporadic. Here, we report a case of bilateral pheochromocytoma (PCC) with a variant in the MYC-associated factor X (*MAX*) gene (c.295 + 1G > A). A male patient was diagnosed with adrenal pheochromocytoma (PCC) and underwent a left adrenalectomy at the age of 40. A new tumor in the right adrenal gland was detected at the age of 43. Urinary metanephrine and normetanephrine concentrations gradually increased. The size of the right adrenal PCC continued to increase one year after detection. Genetic testing of the peripheral blood revealed the presence of a pathogenic variant in *MAX*. The natural history of adrenal PCCs with the *MAX* variant has not yet been clarified, because the number of reported cases is not sufficient. Thus, clinicians should consider a *MAX* variant when they find bilateral or multiple PCCs.

## 1. Introduction

Collectively known as pheochromocytomas/paragangliomas (PPGLs), pheochromocytomas (PCCs) and paragangliomas (PGLs) are catecholamine-producing tumors that arise from chromaffin cells in the adrenal medulla or paraganglia, respectively [1]. Approximately 10% of PCCs were thought to be familial cases [2]. However, approximately 30–40% of PPGLs have been reported as hereditary tumors [3,4]. *RET*, *VHL*, *SDHA*, *SDHB*, *SDHC*, *SDHAF2*, *NF1*, and *TMEM127* contribute to hereditary PPGLs [5]. In 2011, *MAX* variants were identified as one of the causes of hereditary PCCs [5]. We report a case of bilateral adrenal PCC with a germline variant in MYC-associated factor X (*MAX)* gene without a clear family history of PPGLs.

## 2. Case Presentation

**Figure 1:**
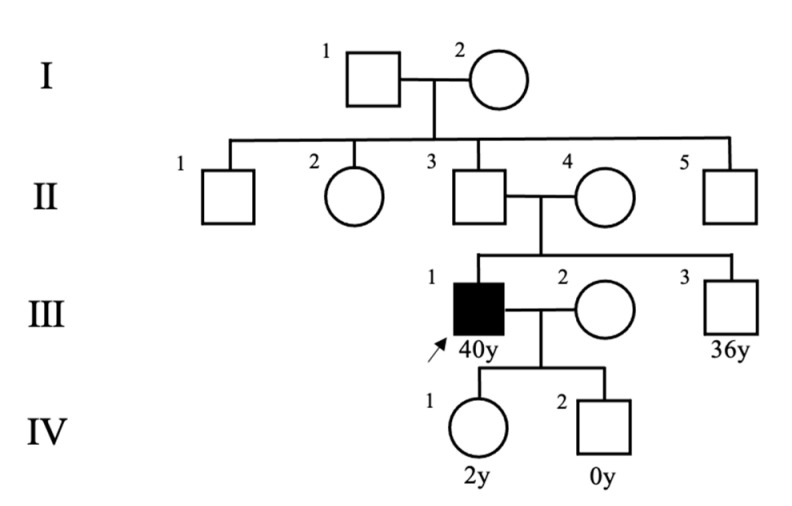
Pedigree of the patient’s family. An arrow indicates the patient.

**Figure 2:**
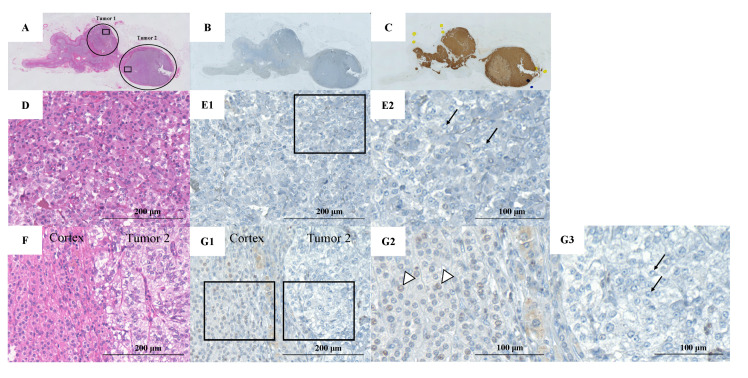
Pathological findings of left adrenal tumors resected from the patient. (**A**) Hematoxylin-eosin (HE) staining of the left adrenal gland (circles show the location of the two tumors); (**B**) MAX staining of the left adrenal gland; (**C**) Chromogranin A staining of the left adrenal gland; (**D**) Hematoxylin and eosin staining of tumor 1 (rectangular area in the tumor 1); (**E1**) MAX staining in tumor 1 (rectangular area in the tumor 1); (**E2**) Rectangular area in panel E1. Pheochromocytoma (PCC) cells in the adrenal medulla show no MAX staining (arrows); (**F**) HE staining of tumor 2 (rectangular area in the tumor 2); (**G1**) MAX staining in tumor 2 (rectangular area in the tumor 2); (**G2**) Left rectangular area in panel G1. Adrenal cortex cells show MAX staining (arrowheads); (**G3**) Right rectangular area in panel G1. The PCC cells show no MAX staining (arrows).

**Figure 3:**
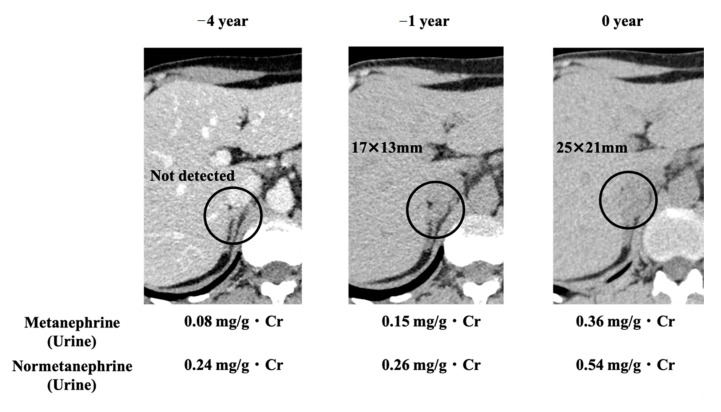
CT images of the right adrenal gland and changes in biochemical test results. Circles indicate right adrenal tumor. Urinary metanephrine and normetanephrine outputs increased with increase in the tumor size.

A healthy 40-year-old man (height, 178 cm; weight, 68 kg) did not have any family history of PPGL, but his father had hypertension. He had a younger brother and two children with no relevant medical history (Figure 1). Two masses in the left adrenal gland, 16 mm (tumor 1 in Figure 2) and 13 mm (tumor 2 in Figure 2), respectively, were incidentally detected by computed tomography imaging during a health check-up program, which are commonly conducted at medical facilities in Japan and other Asian countries in order to identify risk factors and screen for diseases when people are still healthy. His body temperature was 36.5 °C, and pulse rate was 61 beats per minute. Although he had paroxysmal hypertension (up to approximately 200/100 mmHg), his blood pressure was 113/73 mmHg during his first visit to our hospital. Thyroid enlargement and café-au-lait spots on the skin were not observed. Blood and urine tests revealed the following: plasma adrenaline, 74 pg/mL; plasma noradrenaline, 2119 pg/mL; urinary metanephrine, 0.54 mg/day; and urinary normetanephrine, 1.32 mg/day. ^123^I-metaiodobenzylguanidine (MIBG) scintigraphy showed two PCCs in the left adrenal gland. After left adrenalectomy, urinary normetanephrine levels rapidly normalized. The histological pattern of both tumors showed a zellballen and high cellularity (>250 cells/ high power field (HPF)), respectively. Tumor 1 showed absence of vascular and capsular invasion, whereas tumor 2 indicated capsular invasion. The Ki-67 labeling indices of tumor 1 and 2 were 0.1% and 0.6%, respectively. The grading system for adrenal pheochromocytoma and paraganglioma (GAPP) scores of both tumors corresponded to moderately differentiated tumors (tumor 1, score 5; tumor 2, score 3). Three years later, a mass was found in the right adrenal gland. The tumor size increased from 17 to 25 mm in diameter and urinary normetanephrine levels increased from 0.26 mg/g Cr to 0.54 mg/g Cr in one year (Figure 3). ^123^I-MIBG scintigraphy indicated an accumulation in the right adrenal mass. He was diagnosed with right adrenal PCC. Although the family history was unclear, this case was highly suspected of being hereditary PCC because of the presence of bilateral and multiple masses. PCR-direct sequencing of the peripheral blood samples did not show pathological variants in *RET* and *VHL*. Subsequently, the PCR test revealed a heterozygous germline variant in *MAX* (NM_002382.5: c.295 + 1G > A). Immunohistochemistry of the left adrenal tumor tissue with a MAX C-terminus-specific antibody (ab101271, Abcam, Cambridge, United Kingdom) showed no staining of tumor cells in this case (Figure 2). Besides his parents, the patient had a younger brother (adult) and two young children. Despite our recommendation, his family members did not undergo the genetic tests. The patient was treated with an alpha-blocker. Written informed consent was obtained from the patient, and ethical approval for this study was obtained from the Institutional Review Board of Shizuoka General Hospital.

## 3. Discussion

Among all PPGLs, the frequency of hereditary cases was reported to be 33.8% [3,4,6,7,8,9,10,11]. The Endocrine Society clinical practice guidelines state that genetic testing should be considered in all PPGL cases [12]. They also reported that 11–13% of clinically sporadic PCCs are hereditary tumors [13]. For patients, especially those with young, bilateral, multiple, extra-adrenal, or malignant PPGLs, identification of hereditary tumors might be beneficial, because clinical characteristics vary with each genetic background [14]. In this case, we found a germline variant in the *MAX* gene, which was seemingly sporadic. The characteristics of PPGLs with some genetic types, such as *MAX* gene, are difficult to describe because there have been few reports.

A large international study confirmed that *MAX* germline and somatic variants were responsible for PCCs in 1.12% and 1.65% of cases, respectively [15]. One small study, which included eight index patients and three relatives, showed a high penetration rate of 73% for up to 40 years of age, although the rate could be affected by a selection bias [16].

*MAX* is considered a tumor suppressor gene, forms the MYC-MAX-MXD1 network, and acts as a transcription factor that regulates cell proliferation, differentiation, and death [17]. Heterodimerization of MAX with MYC acts as a transcriptional activator, whereas the heterodimers of MAX with MXD1 repress MYC-dependent transcriptional activities by antagonizing the MYC-MAX function [18]. The *MAX* gene comprises five exons. The previously reported variants in *MAX* were distributed along the gene but were particularly frequent in exons 3 and 4, matching some of the crucial residues within the conserved basic helix-loop-helix leucine zipper (bHLH-Zip) domain of *MAX* [15].

**Figure 4:**
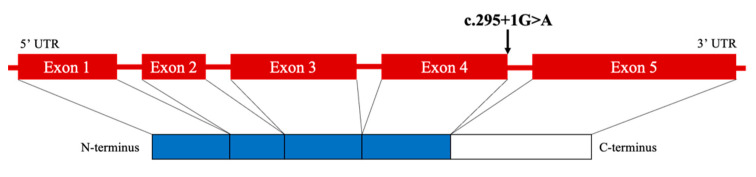
Schematic diagram of *MAX* mutation in the patient. UTR, untranslated region.

The peripheral blood sample of our patient showed a heterozygous single nucleotide substitution in *MAX* (c.295 + 1G > A). The majority of *MAX* mutations result in truncated proteins [15]. A truncated protein was observed in the case of the same variant (c.295 + 1G > A), as reported previously [5]. In that case, the mutation site was in the intron but was located at the donor/acceptor splice site, leading to the skipping of exon 4. Therefore, we estimated that skipping exon 4 produced truncated proteins that had no ability to regulate cell proliferation, differentiation, and apoptosis and promoted the development of tumors in our case (Figure 4).

A Pubmed search was performed using the key terms pheochromocytoma and variant. All searches were limited to reports published in the English language, dating from 1972 to April 2022. Among 499 studies, 213 articles reported variants related to PPGLs and 16 papers reported *MAX* variants. We summarized the characteristics of the cases with PPGLs in *MAX* variants [5,15,16,19,20,21,22,23,24,25,26,27,28,29,30,31] (Supplementary Table S1). Combining data from the 16 reports in *MAX* variants revealed that 42/71 cases (59.2%) had bilateral PCCs, 9/59 cases (8.5%) had PGLs, and 31/70 cases (44.3%) had an apparent family history of PPGLs. In this review, 11/65 patients (16.9%) had metastases. Unlike *SDHB*, *MAX* variants do not appear to be a high risk for malignancy, considering the frequency; however, the reported case of bilateral PCC with a variant in *MAX* (c.295 + 1G > A) had the same variant as our case, and was found to be malignant [5]. Apart from the aforementioned case [5], no other variants were exactly the same. However, another patient with a variant in *MAX* (c.295 + 1G > T) at the same site was presented with metastasis in another report [15]. Our search showed that there were 16 studies reporting *MAX* variants; only eight of them performed immunostaining for MAX. Thus, our report is valuable and contributes to the growing body of knowledge on this field.

In recent years, *MAX* variants have been reported to be associated with endocrine tumors, such as pituitary adenomas (prolactinoma and acromegaly) and parathyroid adenomas in addition to PPGLs [29]. *MAX* variants may also be associated with pancreatic neuroendocrine neoplasms [32]. In our case, hypercalcemia was not observed. There were no physical findings, clinical histories, or examinations suggestive of acromegaly or prolactinoma. Abdominal computed tomography did not exhibit tumors in the pancreas.

Burnichon et al. reported that urinary levels of normetanephrine were elevated in all patients with the *MAX* variant, with no difference between the group with the *VHL* and *SDHB/D* variant or the group with the *RET*/*NF1* variant. In contrast, patients with the *MAX* variant had normal or moderately increased urinary outputs of metanephrine with an intermediate distribution. The metanephrine outputs were higher in the *VHL*/*SDH* group than in the *RET*/*NF1* group [15]. In our case, the urinary metanephrine output was within the normal range but increased moderately compared to that in the *VHL*/*SDH* group. However, urinary normetanephrine output was increased. The urinary biochemical phenotype of our patient was consistent with that report.

Our case showed bilateral and multiple tumors without an apparent family history of PPGLs, and there were no findings suggestive of metastasis at this time. His tumors had typical features of PPGLs in *MAX* variants.

All patients with PPGL are recommended to be engaged in shared decision-making for genetic testing [12]. When a *MAX* pathogenic variant is found, Muth et al. recommended that all adult first-degree relatives be tested through targeted testing of the variant on DNA [33]. However, it is important to respect personal autonomy.

Four years after the first detection of the tumors, there were no findings suggesting metastasis in the case discussed; however, the possibility of malignancy or further tumor development cannot be ruled out. Variant classification according to the American College of Medical Genetics and Genomics Guidelines (ACMG) suggested that the variant was pathogenic, as our case satisfied PVS1, PM2, PP3, and PP4 [34]. Careful follow-up is required in this case.

Recent studies have indicated that the frequency of germline variants of PPGLs is very high among all human tumors. Besides *MAX*, multiple new PPGL-related genes such as *CSDE1*, *H3F3A*, *MET*, *MERTK*, *UBTF-MAML3*, *SLC25A11*, *IRP1*, *DLST*, and *SUCLG2* have been discovered in recent years [35]. With the expansion of our knowledge of genetics, new biomarkers and artificial intelligence can also help assess the metastatic risk and overall prognosis of each individual. This disease is no longer the “ten percent tumor” in terms of genetics. Moreover, certain PPGL cases can promote metastasis. Whenever a case of PPGL is seen, we should consider the possibility of a familial or metastatic case. Finally, the possibility of hereditary PPGL including the *MAX* gene should be considered, particularly in cases of multiple or bilateral PPGLs, even without a clear family history such as in the case of this patient.

## Data Availability

Not applicable.

## Supplementary Materials

The following supporting information can be downloaded at: https://www.mdpi.com/article/10.3390/clinpract12030035/s1, Table S1: Clinical features of PPGL with MAX variants.

## Abbreviations

Sex: F, female; M, male; PCC, adrenal pheochromocytoma; bPCC, bilateral adrenal pheochromocytoma; mPCC, multiple adrenal pheochromocytoma; PGL, paraganglioma. Other diseases: BrC, breast cancer; RO, renal oncocytoma; SCCT, squamous cell carcinoma of the tongue; CCH, C-cell hyperplasia; PA, pituitary adenoma; HPT, hyperparathyroidism; ReC, renal carcinoma; β-T, β-thalassemia; T1DM, type 1 diabetes; PRLoma, prolactinoma; Acro, acromegaly; TC, thyroid cancer; GNB, ganglio-neuroblastoma; NB, neuroblastoma; RiC, rib chondrosarcoma; LA, lung adenocarcinoma; IHC, immunohistochemistry. IHC: Pos, positive; Neg, negative.
