# Supplementary material for: Bilateral Pheochromocytoma with Germline MAX Variant without Family History
Source: Clin Pract. 2022 May 7;12(3):299–305. doi: 10.3390/clinpract12030035 (PMC9149808; doi:10.3390/clinpract12030035)
Supplement: Supplementary file 1 [file clinpract-12-00035-s001.zip › clinpract-1702561-supplementary.pdf]

Table S1

Reference	Sex	Age	Family History	PCC	PGL	Metastasis	Other Disease	cDNA Variant	Protein Alteration	IHC
Comino-Méndez [5]	M	29	Yes	bPCC	-	No	-	c.223C>T	p.(Arg75X)	-
	M	35	Yes	bPCC	-	No	-	c.223C>T	p.(Arg75X)	-
	F	34	Yes	bPCC	-	No	-	c.223C>T	p.(Arg75X)	-
	F	28	Yes	bPCC	-	No	-	c.223C>T	p.(Arg75X)	-
	M	32	Yes	bPCC	-	Yes	-	c.295+1G>A	p.?	-
	F	46	Yes	bPCC	-	Yes	-	c.1A>G	p.(Met1?)	-
	M	29	Yes	PCC	-	No	-	c.1A>G	p.(Met1?)	-
	M	17	No	bPCC	-	No	-	c.97C>T	p.(Arg33X)	-
	F	47	Yes	bPCC	-	No	-	c.185_186delA	p.(Gln62Asn*fs*X23)	-
	F	26	No	PCC	-	Yes	-	c.67G>A	p.(Asp23Asn)	-
	F	22	No	PCC	-	No	-	c.425C>T	p.(Ser142Leu)	-
	F	41	Yes	PCC	-	No	-	c.281T>C	p.(Leu94Pro)	-
Burnichon [15]	M	27	No	PCC	No	No	No	c.1-?_483.?del	p.?	Neg
	F	46	No	bPCC	Yes	No	No	c.2T>A	p.?	Neg
	F	43	No	bPCC	Yes	No	BrC, RO	c.73C>T	p.(Arg25Trp)	-
	M	23	Yes	bPCC	No	No	No	c.97C>T	p.(Arg33)	-
	M	27	Yes	bPCC	No	No	No	c.97C>T	p.(Arg33)	-
	M	34	No	bPCC	No	No	No	c.97C>T	p.(Arg33)	Neg
	F	58	Yes	PCC	No	No	No	c.97C>T	p.(Arg33)	Neg
	F	26	Yes	PCC	No	No	No	c.97C>T	p.(Arg33)	Neg
	M	38	No	PCC	No	No	SCCT	c.97C>T	p.(Arg33)	-
	M	24	Yes	bPCC	No	No	CCH	c.97C>T	p.(Arg33)	-
	F	43	No	PCC	Yes	No	No	c.97C>T	p.(Arg33)	-
	F	18	No	bPCC	No	No	No	c.171+1G>A	p.?	-
	F	55	No	bPCC	No	No	No	c.178C>T	p.(Arg60Trp)	-
	F	34	No	bPCC	No	No	No	c.212T>G	p.(Ile71Ser)	Pos
	M	57	No	PCC	No	No	PA	c.220A>G	p.(Met74Val)	Pos
	M	18	No	bPCC	No	No	HPT	c.223C>T	p.(Arg75)	-
	F	18	Yes	PCC	No	Yes	No	c.244C>T	p.(Gln82)	-
	F	40	No	bPCC	Yes	Yes	No	c.295+1G>T	p.?	Neg
	M	13	Yes	PCC	No	No	No	c.305T>C	p.(Leu102Pro)	-
	F	48	No	No	Yes	No	No	c.-18C>T	p.(=)	-
	M	13	No	PCC	Yes	No	No	c.25G>T	p.(Val9Leu)	Pos
	M	22	No	PCC	No	No	No	c.63G>T	p.(=)	-

	F	80	No	PCC	No	No	No	c.414G>A	p.(=)	-
	F	29	No	PCC	No	No	No	c.269G>C	p.(Arg90Pro)	-
	M	39	No	PCC	No	No	No	c.223C>T	p.(Arg75)	Neg
	F	57	No	PCC	No	No	ReC	c.103C>T	p.(Arg35Cys)	Pos
	M	24	No	PCC	No	No	No	c.140_157del	p.(Arg47_Ser52del)	Neg
	F	56	No	PCC	No	No	No	c.25del	p.(Val9Trpfs56)	Neg
Korpershoek [20]	M	45	Yes	bPCC	No	No	RO, β-T	-	-	Neg
	M	28	Yes	bPCC	No	Yes	β-T, T1DM	-	-	Neg
	M	55	Yes	bPCC	No	No	No	-	-	Neg
Bausch [16]	F	36	No	PCC	No	-	-	c.73C>T	-	-
	F	23	No	PCC	No	-	-	c.146C>G	-	-
	F	50	Yes	bPCC	No	-	-	c.242_243del	-	-
	M	21	No	PCC	No	-	-	c.292dup	-	-
	M	26	No	bPCC	No	-	-	c.307G>T	-	-
Roszko [21]	F	49	No	bPCC	No	No	PRLoma, HPT	c.296-1G>T	-	Neg
Shibata [19]	F	24	No	bPCC	Yes	No	No	c.70_73 del	p.(Lys24fs*40)	-
Romanet [22]	M	27	Yes	bPCC	No	No	No	c.97C>T	p.(Arg33*)	Neg
Daly [23]	M	32	No	PCC	No	No	PRLoma	-	-	Neg
	F	35	No	bPCC	No	No	Acro, TC	-	-	Neg
	M	22	No	bPCC	No	No	Acro	-	-	Neg
Kobza [24]	F	39	No	bPCC	No	No	PRLoma, HPT	c.171+2T>A	-	-
Pozza [25]	F	15	No	bPCC	Yes	Yes	GNB	c.299G>C	p.(Arg100Pro)	-
Chang [26]	F	31	No	PCC	No	Yes	TC	c.97C>T	p.(Arg33Ter)	Neg
Choi [27]	F	39	-	bPCC	No	No	-	c.3G>A	p.(Met1?)	-
Duarte [28]	M	27	Yes	PCC	No	No	NB	c.97C>T	p.(Arg33Ter)	-
	M	32	Yes	bPCC	No	No	No	c.97C>T	-	-
	M	24	Yes	bPCC	No	No	No	c.97C>T	-	-
	M	19	Yes	PCC	No	No	No	c.97C>T	-	-
Seabrook [29]	M	21	Yes	bPCC	Yes	Yes	Acro	c.200C>A	p.(Ala67Asp)	Neg
	M	42	Yes	bPCC	No	No	No	c.200C>A	p.(Ala67Asp)	Neg
	M	50	Yes	PCC	No	Yes	No	c.200C>A	p.(Ala67Asp)	Neg
	F	23	Yes	bPCC	No	?	No	c.200C>A	p.(Ala67Asp)	Neg
	M	30	Yes	bPCC	No	No	No	c.200C>A	p.(Ala67Asp)	Neg
	F	14	Yes	bPCC	No	No	Acro	c.200C>A	p.(Ala67Asp)	Neg
	F	21	No	bPCC	No	Yes	RiC, PRLoma, PHT, LA	C.22G>T	p.(Glu8*)	-
Mamedova [30]	F	21	No	bPCC	No	No	PRLom, Acro	c.223C>T	p.(R75X)	-

Lam-Chung [31]	F	38	No	PCC	No	No	No	c.64-2A>G	-	-
